# Mitochondrial membrane transporters as attractive targets for the fermentative production of succinic acid from glycerol in *Saccharomyces cerevisiae*

**DOI:** 10.1093/femsyr/foae009

**Published:** 2024-04-08

**Authors:** Toni Rendulić, Andreea Perpelea, Juan Paulo Ragas Ortiz, Margarida Casal, Elke Nevoigt

**Affiliations:** School of Science, Constructor University, Campus Ring 1, 28759 Bremen, Germany; Centre of Molecular and Environmental Biology, Department of Biology, University of Minho, Campus de Gualtar, 4710-057 Braga, Portugal; Institute of Science and Innovation for Bio-Sustainability (IB-S), University of Minho, Campus de Gualtar, 4710-057 Braga, Portugal; School of Science, Constructor University, Campus Ring 1, 28759 Bremen, Germany; School of Science, Constructor University, Campus Ring 1, 28759 Bremen, Germany; Centre of Molecular and Environmental Biology, Department of Biology, University of Minho, Campus de Gualtar, 4710-057 Braga, Portugal; Institute of Science and Innovation for Bio-Sustainability (IB-S), University of Minho, Campus de Gualtar, 4710-057 Braga, Portugal; School of Science, Constructor University, Campus Ring 1, 28759 Bremen, Germany

**Keywords:** succinate, mitochondria, membrane transporter, yeast, metabolic engineering, mpc3; sdh1

## Abstract

Previously, we reported an engineered *Saccharomyces cerevisiae* CEN.PK113-1A derivative able to produce succinic acid (SA) from glycerol with net CO_2_ fixation. Apart from an engineered glycerol utilization pathway that generates NADH, the strain was equipped with the NADH-dependent reductive branch of the TCA cycle (rTCA) and a heterologous SA exporter. However, the results indicated that a significant amount of carbon still entered the CO_2_-releasing oxidative TCA cycle. The current study aimed to tune down the flux through the oxidative TCA cycle by targeting the mitochondrial uptake of pyruvate and cytosolic intermediates of the rTCA pathway, as well as the succinate dehydrogenase complex. Thus, we tested the effects of deletions of *MPC1, MPC3, OAC1, DIC1, SFC1*, and *SDH1* on SA production. The highest improvement was achieved by the combined deletion of *MPC3* and *SDH1*. The respective strain produced up to 45.5 g/L of SA, reached a maximum SA yield of 0.66 g_SA_/g_glycerol_, and accumulated the lowest amounts of byproducts when cultivated in shake-flasks. Based on the obtained data, we consider a further reduction of mitochondrial import of pyruvate and rTCA intermediates highly attractive. Moreover, the approaches presented in the current study might also be valuable for improving SA production when sugars (instead of glycerol) are the source of carbon.

## Teaser

A novel metabolic engineering strategy targeting mitochondrial membrane transporters and enzymes was successfully applied to significantly improve succinic acid yield in *Saccharomyces cerevisiae*.

## Introduction

Succinic acid (SA) is an industrially relevant compound that can serve as a precursor in the synthesis of numerous speciality and commodity chemicals such as 1,4-butanediol, gamma-butyrolactone, and tetrahydrofuran (Werpy and Petersen 2004). Moreover, SA is utilized as a precursor in biopolymer production and as an additive in food and pharmaceutical products (Ahn et al. 2016). Currently, SA is mostly produced from non-renewable fossil fuel derivatives, namely n-butane (Cok et al. 2014). However, being an important constituent of cellular metabolism, SA can also be produced from renewable carbon via microbial fermentation (Ahn et al. 2016).

The current biotechnological SA production processes rely on fungal and bacterial cell factories (Ahn et al. 2016). In comparison to bacterial processes, the usage of fungal cell factories is advantageous because the latter can produce SA at low extracellular pH, thus enabling a much simpler and cheaper product purification (Jansen and van Gulik 2014, Mancini et al. 2022). Among acid-tolerant fungal organisms, the yeast *Saccharomyces cerevisiae* is especially attractive because it allows extensive metabolic engineering endeavours aiming at maximum yield, productivity, and titre (Abbott et al. 2009).

**Figure 1.**
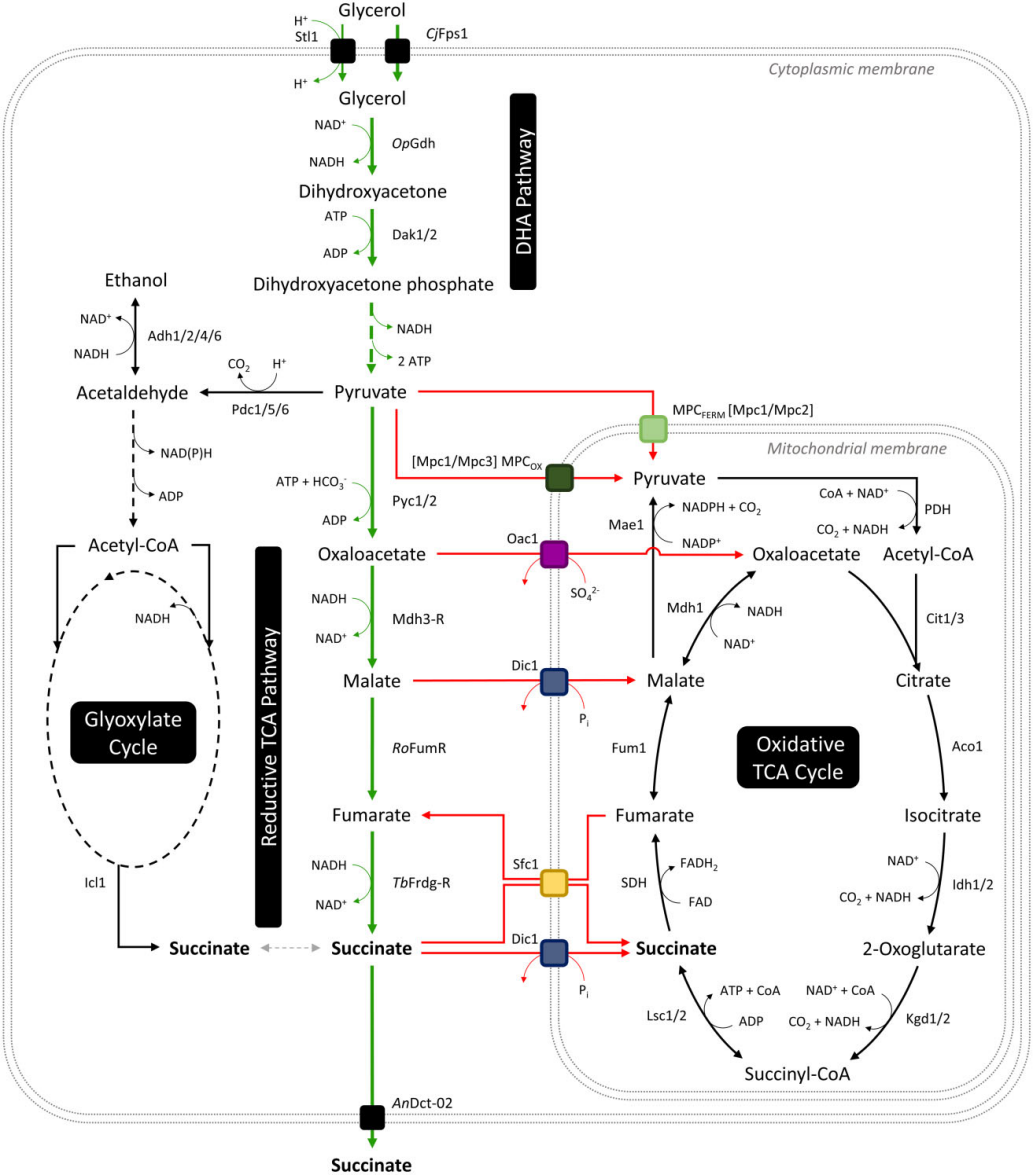
The route for the envisaged succinic acid production from glycerol and CO_2_ (green) alongside other relevant metabolic routes. Mitochondrial transport reactions hypothesized to compete for pyruvate and rTCA intermediates in engineered *S. cerevisiae* strains are highlighted in red. Stl1 glycerol/H^+^ symporter, *Cj*Fps1 aquaglyceroporin from *Cyberlindnera jadinii, Op*Gdh glycerol dehydrogenase from *Ogataea parapolymorpha*, Dak1/2 dihydroxyacetone kinase, Pyc1/2 pyruvate carboxylase, Mdh3-R peroxisomal malate dehydrogenase targeted to the cytosol, *Ro*FumR fumarase from *Rhizopus oryzae, Tb*Frdg-R glycosomal fumarate reductase from *Trypanosoma brucei* retargeted to the cytosol, *An*Dct-02 dicarboxylic acid transporter from *Aspergillus niger*, MPC_OX_ respiratory variant of mitochondrial pyruvate carrier complex, MPC_FERM_ fermentative variant of the mitochondrial pyruvate carrier complex, Oac1 oxaloacetate carrier, Dic1 dicarboxylate carrier, Sfc1 succinate-fumarate carrier, PDH pyruvate dehydrogenase complex, Cit1/3 citrate synthase, Aco1 aconitase, Idh1/2 isocitrate dehydrogenase, Kgd1/2 alpha-ketoglutarate dehydrogenase, Lsc1/2 succinyl-CoA ligase, Fum1 fumarase, SDH succinate dehydrogenase complex, Mdh1 malate dehydrogenase, Mae1 malic enzyme, Pdc1/5/6 pyruvate decarboxylase, Adh1/2/4/6 alcohol dehydrogenase, and Icl1 isocitrate lyase.

Among the microbial metabolic pathways that can generate SA, the reductive branch of the TCA cycle (rTCA pathway) is more attractive than the oxidative branch of the TCA cycle or the glyoxylate cycle because it allows net CO_2_ fixation (e.g. via pyruvate carboxylation; Fig. 1) and thus the highest theoretical SA yield (Ahn et al. 2016). The rTCA pathway requires 2 cytosolic NADH molecules to generate 1 SA molecule from 1 molecule of pyruvate (Fig. 1). This implies that the usage of glycerol and CO_2_ as carbon sources enables a redox-neutral SA production with a 16.67% higher maximum theoretical SA yield compared to the use of glucose and CO_2_ (1.33 Cmol/Cmol compared to 1.14 Cmol/Cmol, respectively) (Malubhoy et al. 2022). Moreover, twice the amount of CO_2_ can be fixed per mol of SA when glycerol is used as the substrate (Steiger et al. 2017).

Notably, many wild-type *S. cerevisiae* strains, including the CEN.PK family, do not utilize glycerol as a sole carbon source in synthetic medium (Xiberras et al. 2019). The growth defect of CEN.PK could be overcome by adaptive laboratory evolution and reverse engineering, as previously demonstrated (Ochoa-Estopier et al. 2011, Ho et al. 2017). In fact, the respective CEN.PK strain only required single point mutations in two genes (*GUT1* and *UBR2*) in order to become a glycerol-utilizing strain with a moderate maximal growth rate in synthetic glycerol media (Ho et al. 2017). Using a different strain background, Klein et al. (2016) demonstrated that the expression of a heterologous aquaglyceroporin (e.g. Fps1 from *Cyberlindnera jadinii*) also had a positive effect on glycerol utilization in *S. cerevisiae*.

An additional challenge, that is particularly relevant for converting glycerol to fermentation products whose formation requires cytosolic NADH (such as SA), is that glycerol metabolism in *S. cerevisiae* is respiratory. In fact, the endogenous glycerol catabolic pathway (L-G3P pathway) in *S. cerevisiae* transfers a part of the electrons from glycerol to FADH_2_ and subsequently to oxygen via the respiratory chain. By abolishing the FAD-dependent L-G3P pathway and establishing a heterologous so-called DHA pathway, the respective electrons can be trapped in cytosolic NADH molecules (Klein et al. 2016). This pathway replacement is a key prerequisite for redox-neutral SA production (Fig. 1). Efficient SA production (reaching up to 47% of the maximum theoretical yield) from glycerol and CO_2_ has been achieved by equipping a *S. cerevisiae* DHA pathway derivative of CEN.PK113-1A (also carrying a deleted *GUT1* gene, a functional *UBR2* allele, and the Fps1 from *C. jadinii*) with a heterologous rTCA pathway and a heterologous SA exporter (Xiberras et al. 2020, Malubhoy et al. 2022). The best-performing strain has also been demonstrated to achieve a slight net fixation of CO_2_ during the phase of SA production (Malubhoy et al. 2022).

Even so, the results presented in the study of Malubhoy et al. (2022) indicated that a significant amount of carbon still entered the CO_2_-releasing oxidative TCA cycle affecting the total CO_2_ net balance during the fermentation process in an unwanted manner. It is clear that oxTCA is necessary for ATP generation and the supply of precursors for biomass formation. However, one might wonder whether the activity of this pathway exceeded the level required for maintenance and growth. It must be noted in this context that *S. cerevisiae* naturally upregulates its respiratory metabolism when glycerol is the sole carbon source (Xiberras et al. 2019).

During respiratory metabolism, a part of the carbon is assumed to enter the mitochondria in the form of pyruvate, which is converted to acetyl-CoA by the pyruvate dehydrogenase complex (Fig. 1). Moreover, it is expected that carbon enters the mitochondria in the form of cytosolic rTCA pathway intermediates such as oxaloacetate, malate, fumarate, and even succinate. In fact, these compounds are assumed to enter the mitochondria via different membrane transporters (see below). Inside the mitochondria, the additional C_4_ compounds can either help in anabolic reactions and/or eventually result in the formation of pyruvate from malate via the malic enzyme (Fig. 1), which eventually leads to the complete oxidation into CO_2_ via the PDH complex and the oxidative branch of the TCA cycle. In that regard, several transporters located in the inner mitochondrial membrane (Palmieri et al. 2006, Ferramosca and Zara 2021) represent promising targets in order to fine-tune the flux of carbon into the mitochondria.

The mitochondrial pyruvate carrier (MPC) complex is responsible for the uptake of cytosolic pyruvate into the mitochondria of *S. cerevisiae*, where it is dissimilated by the pyruvate dehydrogenase complex and the oxidative TCA cycle for energy production or utilized as a precursor in the biosynthesis of branched-chain amino acids (Herzig et al. 2012) (Fig. 1). Two functional variants of the MPC complex exist (Bender et al. 2015). The MPC_FERM_ is a heterodimer formed by the Mpc1 and Mpc2 subunits responsible for pyruvate uptake during fermentative growth. The MPC_OX_ is a heterodimer formed by the Mpc1 and Mpc3 subunits, and it is responsible for pyruvate uptake during growth on non-fermentable carbon sources. Among the two variants, the MPC_OX_ has been shown to have a significantly higher pyruvate uptake activity, thereby promoting the dissimilation of pyruvate via the oxidative TCA cycle under the respective conditions (Bender et al. 2015). Thus, the *MPC3* gene is considered to be a promising deletion target with the goal of increasing the supply of cytosolic pyruvate for SA production when cells grow on glycerol. In the study of Bender et al. (2015), the deletion of the *MPC1* gene completely abolished the mitochondrial pyruvate uptake. Notably, this might not lead to complete growth impairment of our SA-producing strain equipped with the rTCA pathway due to the import of cytosolic C_4_ compounds by other mitochondrial transporters (as described below) and their potential conversion to mitochondrial pyruvate via the malic enzyme (Mae1) (Boles et al. 1998). Thus, *MPC1* was also considered a promising target gene.

*Saccharomyces cerevisiae* has two mitochondrial transporters which enable the net uptake of rTCA intermediates and SA into the mitochondria—Oac1 and Dic1 (Ferramosca and Zara 2021). Oac1 mediates oxaloacetate import by exchanging cytosolic oxaloacetate with mitochondrial sulfate (Palmieri et al. 1999), while Dic1 mediates malate and SA import by exchanging cytosolic malate or SA with mitochondrial phosphate (Palmieri et al. 1996) (Fig. 1). The main physiological role of these two transporters is anaplerotic, i.e. replenishing the mitochondrial TCA cycle intermediates spent in anabolic reactions (Ferramosca and Zara 2021). By deleting one of the two transporters, the mitochondrial consumption of cytosolic oxaloacetate, malate, and SA could be directly prevented. Indirectly, this may also tune down the overall activity of the oxidative TCA cycle by limiting the process of anaplerosis.

Sfc1 is another mitochondrial transporter with affinity for rTCA intermediates, primarily exchanging cytosolic SA with mitochondrial fumarate (Palmieri et al. 1997). In contrast to Oac1 and Dic1, the activity of Sfc1 does not lead towards net mitochondrial consumption of C_4_ dicarboxylic acids since it exchanges one C_4_ molecule with another. However, in *S. cerevisiae* strains equipped with the rTCA pathway, the activity of Sfc1 in conjunction with cytosolic fumarate reductase and mitochondrial succinate dehydrogenase may lead towards a wasteful cycle in which the electrons from cytosolic NADH are transferred to mitochondrial FAD and end up in the respiratory chain (Fig. 1), which is counterproductive for the NADH-dependent cytosolic SA production.

The SDH complex plays an important role in the hypothesized mitochondrial consumption of cytosolic SA via Dic1 and in the hypothesized transfer of electrons into the respiratory chain via Sfc1 (Fig. 1). Thus, *SDH1* deletion represents an alternative approach to tackle these issues by disrupting the mitochondrial conversion of SA into fumarate, and it may also serve as a control to better interpret the results obtained from the *DIC1* and *SFC1* deletions.

In this work, we individually deleted the genes *MPC1, MPC3, OAC1, DIC1*, and *SFC1* encoding the aforementioned mitochondrial transporters as well as the *SDH1* gene with an aim to increase SA production via the rTCA pathway in *S. cerevisiae*. We performed shake-flask cultivations of the respective deletion mutants and compared the effects of these deletions on succinic acid production, glycerol consumption, biomass formation, and byproduct formation.

## Materials and methods

### Strains, plasmids, and strain maintenance

The *S. cerevisiae* strains and plasmids used in this study are listed in Tables 1 and 2, respectively. Yeast cells were routinely grown on solid YPD media, which contained yeast extract (10 g/L), peptone (20 g/L), glucose (20 g/L), and agar (20 g/L). Agar plates were cultivated in a static incubator at 30°C. Media were supplemented with phleomycin (20 mg/L), hygromycin B (300 mg/L), or nourseothricin (100 mg/L) for selection purposes when needed. *Escherichia coli* DH5α cells were used for plasmid isolation and were routinely grown in lysogeny broth (LB) containing NaCl (10 g/L), yeast extract (5 g/L), and peptone (10 g/L) adjusted to a pH of 7.5 with 2 M NaOH (Bertani 1951). For the selection and maintenance of plasmid-containing cells, ampicillin (100 mg/L) was added. Cultivations were performed on an orbital shaker at 250 rpm and 37°C and plasmids were isolated using the GeneJET^TM^ Plasmid Miniprep Kit (Thermo Fisher Scientific, Waltham, MA, USA).

**Table 1.** *Saccharomyces cerevisiae* strains used in this study.

Strain name	Description	Reference
UBR2_CBS_-DHA-SA-AnDCT-02 (2)	CEN.PK113-1A *ubr2::UBR2_CBS 6412–13A_; gut1::loxP-ble-loxP; YGLCτ3(8)::P_TDH3_-CjFPS1-T_RPL15A_-P_TEF1_-Opgdh-T_CYC1_-P_ADH2_-ScDAK1-T_TPS1_; YPRCτ3(21)::P_JEN1_-ScMDH3_rt_-T_IDP1_-P_HOR7_-RoFUM-T_DIT1_-P_FBA1_-TbFRD_rt_-T_ADH1_-P_COX7_-AnDCT-02-T_CYC1_*	Malubhoy et al. (2022)
DHA-SAT	CEN.PK113-1A *ubr2::UBR2_CBS 6412–13A_; gut1::loxP; YGLCτ3(8)::P_TDH3_-CjFPS1-T_RPL15A_-P_TEF1_-Opgdh-T_CYC1_-P_ADH2_-ScDAK1-T_TPS1_; YPRCτ3(21)::P_JEN1_-ScMDH3_rt_-T_IDP1_-P_HOR7_-RoFUM-T_DIT1_-P_FBA1_-TbFRD_rt_-T_ADH1_-P_COX7_-AnDCT-02-T_CYC1_* Derived from the strain UBR2_CBS_-DHA-SA-AnDCT-02 (2) by removing the phleomycin resistance marker (*ble*) from the *gut1* locus via Cre/loxP recombinase	This study
*mpc3*Δ	DHA-SAT *mpc3::loxP-ble-loxP*	This study
*mpc1*Δ	DHA-SAT *mpc1::loxP-ble-loxP*	This study
*oac1*Δ	DHA-SAT *oac1::loxP-ble-loxP*	This study
*dic1*Δ	DHA-SAT *dic1::loxP-ble-loxP*	This study
*sfc1*Δ	DHA-SAT *sfc1::loxP-ble-loxP*	This study
*sdh1*Δ	DHA-SAT *sdh1::loxP-ble-loxP*	This study
*PYC2_oe_*	CEN.PK113-1A *ubr2::UBR2_CBS 6412–13A_; gut1::loxP-ble-loxP; YGLCτ3(8)::P_TDH3_-CjFPS1-T_RPL15A_-P_TEF1_-Opgdh-T_CYC1_-P_ADH2_-ScDAK1-T_TPS1_; YPRCτ3(21)::P_JEN1_-ScMDH3_rt_-T_IDP1_-P_HOR7_-RoFUM-T_DIT1_-P_FBA1_-TbFRD_rt_-T_ADH1_-P_COX7_-AnDCT-02-T_CYC1_; XI-3::P_HOR7_-ScPYC2-T_TPS1_* Derived from the strain UBR2_CBS_-DHA-SA-AnDCT-02 (2) via genomic integration of the *P_HOR7_-ScPYC2-T_TPS1_*expression cassette into the *XI-3* locus	Malubhoy et al. (2022)
*PYC2_oe_-mpc3*Δ	*PYC2_oe_ mpc3::hphMX*	This study
*PYC2_oe_-dic1*Δ	*PYC2_oe_ dic1::hphMX*	This study
*PYC2_oe_-sdh1*Δ	*PYC2_oe_ sdh1::hphMX*	This study
*PYC2_oe_-mpc3*Δ *sdh1*Δ	*PYC2_oe_-mpc3*Δ *sdh1::loxP-natMX-loxP*	This study

**Table 2.** Plasmids used in this study.

Plasmid name	Description	Reference
pNatCre	Used for the removal of *loxP-ble-loxP* disruption cassettes from the genome. Contains the Cre-recombinase gene under the control of the *GAL1* promoter and the nourseothricin resistance marker for selection.	Steensma and Ter Linde (2001)
pUG66	Template for the amplification of *loxP-ble-loxP* disruption cassettes.	Gueldener et al. (2002)
pAG32	Template for the amplification of *hphMX* disruption cassettes.	Goldstein and McCusker (1999)
pUG74	Template for the amplification of *loxP-natMX-loxP* disruption cassettes.	Hegemann and Heick (2011)

### Marker rescue via the Cre-loxP system

To remove the phleomycin resistance marker (*ble*) flanked by loxP sites from the genome of *S. cerevisiae* strains, the respective strains were transformed with pNatCre (Table 2) according to the lithium acetate method described by Gietz et al. (1995). Positive transformants were selected on a solid YPD medium supplemented with nourseothricin. A single colony of the transformed strain was inoculated into 3 mL of YPD medium supplemented with nourseothricin and grown at 30°C and 200 rpm overnight. Cells were then centrifuged at 800 g for 5 min and washed 3 times in 5 mL of YPGal medium, which contained yeast extract (10 g/L), peptone (20 g/L), and galactose (20 g/L). The obtained pellet was resuspended in 3 mL of YPGal supplemented with nourseothricin (100 mg/L) and incubated at 30°C and 200 rpm for 6–8 h. Afterwards, an aliquot of the cell suspension (10 µL) was spread on a solid YPD medium and incubated at 30°C for 2 days. Single colonies were streaked in a lawn on solid YPD media with and without phleomycin. In order to verify the loss of the *ble* marker via diagnostic PCR, genomic DNA was isolated from those clones that were not capable of growing in the presence of phleomycin. The pNatCre plasmid was subsequently removed from the verified clone by serial transfers in YPD medium lacking nourseothricin.

### Gene deletion

Gene deletions were obtained by using disruption cassettes consisting of the phleomycin or nourseothricin resistance markers flanked by loxP sites (*loxP-ble-loxP* or loxP-natMX-loxP, respectively) or the hygromycin resistance marker (*hphMX*) (Supplementary Table S1). The disruption cassettes were amplified from pUG66, pUG74, or pAG32 plasmids, respectively, using the primer pairs listed in Supplementary Table S1. The primers used for amplification contained at their 5' terminal end a 40–60 bp sequence complementary to the region immediately upstream or downstream of the start or stop codon of the gene to be deleted. Preparative PCRs for amplification of disruption cassettes were performed using Phusion® High-Fidelity DNA Polymerase (New England BioLabs, Frankfurt am Main, Germany). PCR conditions were adapted to the guidelines of the manufacturer. PCR products were purified by using the GeneJET^TM^ PCR Purification Kit (Thermo Fisher Scientific). Transformation of *S. cerevisiae* with linear disruption cassettes was performed according to the lithium acetate method described by Gietz et al. (1995) using 2 µg of DNA for one transformation. Positive transformants were selected on solid YPD media supplemented with the appropriate antibiotic.

### Isolation of genomic DNA from *S. cerevisiae* transformants and diagnostic PCR

Both correct integration of a disruption cassette into the genome of *S. cerevisiae* and successful marker removal were verified by diagnostic PCR using OneTaq Quick-load DNA polymerase and buffer according to the manufacturer’s guidelines (New England Biolabs). Genomic DNA from *S. cerevisiae* strains was isolated according to a modified protocol from Hoffman and Winston (1987). Approximately 50 mg of cells were suspended in 200 µL of TE buffer (10 mM Tris, 1 mM EDTA, pH 8.0). Subsequently, 300 mg of acid-washed glass beads (diameter of 0.425–0.6 mm) and 200 µL of phenol:chloroform:isoamyl alcohol (25:24:1) were added. The tubes were vortexed at maximum speed for 2 min and centrifuged at 15 700 g for 10 min. The aqueous phase (1 µL) was used as a template in 25 µL PCR reactions. Primers for diagnostic PCR were designed to bind within the disruption cassette and/or within the genomic DNA upstream and downstream of the sequence to be deleted.

### *Saccharomyces cerevisiae* strain construction

The DHA-SAT strain was obtained by removing the phleomycin resistance marker (*ble*) from the *gut1* locus in the UBR2_CBS_-DHA-SA-AnDCT-02 (2) strain via Cre-recombinase. The *mpc3*Δ, *mpc1*Δ, *oac1*Δ, *dic1*Δ, *sfc1*Δ, and *sdh1*Δ strains were obtained by deleting the respective genes in the DHA-SAT strain using *loxP-ble-loxP* disruption cassettes (Supplementary Table S1). The *PYC2_oe_-mpc3*Δ, *PYC2_oe_-dic1*Δ, and *PYC2_oe_-sdh1*Δ strains were obtained by deleting the respective genes in the *PYC2_oe_* strain using *hphMX* disruption cassettes (Supplementary Table S1). The *PYC2_oe_-mpc3*Δ *sdh1*Δ strain was obtained by deleting the *SDH1* gene in the *PYC2_oe_-mpc3*Δ strain using a *loxP-natMX-loxP* disruption cassette.

### Media and cultivation conditions for the shake-flask cultivations to test the production of succinic acid from glycerol

Synthetic medium containing glucose (20 g/L) and ammonium sulphate (5 g/L) as the carbon and nitrogen sources, respectively, was used for all precultures and intermediate cultures. The synthetic medium was prepared according to Verduyn et al. (1992), containing 3 g/L KH_2_PO_4_, 0.5 g/L MgSO_4_·7H_2_O, 15 mg/L EDTA, 4.5 mg/L ZnSO_4_·7H_2_O, 0.84 mg/L MnCl_2_·2H_2_O, 0.3 mg/L CoCl_2_·6H_2_O, 0.3 mg/L CuSO_4_·5H_2_O, 0.4 mg/L NaMoO_4_·2H_2_O, 4.5 mg/L CaCl_2_·2H_2_O, 3 mg/L FeSO_4_·7H_2_O, 1 mg/L H_3_BO_3_, and 0.1 mg/L KI. After heat sterilization of the medium, appropriate aliquots of autoclaved glucose solution (50% w/v) and filter-sterilized vitamins were added. Final vitamin concentrations were: 0.05 mg/L D-(+)-biotin, 1 mg/L D-pantothenic acid hemi-calcium salt, 1 mg/L nicotinic acid, 25 mg/L myo-inositol, 1 mg/L thiamine chloride hydrochloride, 1 mg/L pyridoxine hydrochloride, and 0.2 mg/L 4-aminobenzoic acid. The pH of the synthetic glucose medium was adjusted to 6.5 with 4 M KOH. All main experiments for assessing succinic acid production in shake flask batch cultivations were performed in a synthetic medium containing 60 mL/L (75.6 g/L) glycerol as the sole carbon source, urea (2.27 g/L) as the nitrogen source, and CaCO_3_ (30 g/L), according to Malubhoy et al. (2022). To prepare this latter medium, an appropriate aliquot of a urea stock solution (113.5 g/L) was added to obtain a final concentration of 2.27 g/L. The pH of the CaCO_3_-buffered synthetic glycerol medium was adjusted to 6.0 with 4 M KOH and the medium was then filter-sterilized. CaCO_3_ was added as a sterile powder only after inoculation, as described below.

For pre-cultivation, cells from a single colony were used to inoculate 3 mL of the synthetic glucose medium in a 10 mL glass tube and incubated at orbital shaking of 200 rpm and 30°C overnight. The pre-culture was used to inoculate 10 mL of the same medium in a 100-mL Erlenmeyer flask (closed with a metal cap), adjusting an OD_600_ of 0.2. This culture, hereafter referred to as intermediate culture, was cultivated under the same conditions for 24 h. The appropriate culture volume from the intermediate culture (in order to later adjust an OD_600_ of 0.2 in 100 mL of synthetic glycerol medium) was centrifuged at 800 g for 5 min and the supernatant was discarded. The cell pellet was then washed once by re-suspending the cells in the synthetic glycerol medium. The cell suspension was centrifuged again and re-suspended in 100 mL of the same medium, adjusting to a final OD_600_ of 0.2. Subsequently, the entire cell suspension was added to a sterile 500-mL Erlenmeyer flask already containing 3 g of sterile CaCO_3_. The Erlenmeyer flasks were closed with cotton plugs. The main cultures were incubated by orbital shaking of 200 rpm and 30°C. Samples for OD_600_ determination and HPLC analysis were taken at regular time intervals. For OD_600_ measurements, samples were diluted in 0.2 M HCl, ensuring complete dissolving of the suspended CaCO_3_.

### Metabolite analysis by HPLC

Samples of culture supernatants (1 mL) were first filtered through 0.2 mm Minisart RC membrane filters (Sartorius, Göttingen, Germany) and stored at −20°C until analysis. The concentrations of succinic acid, malic acid, glycerol, and ethanol in culture media were determined using a Waters HPLC system (Eschborn, Germany) consisting of a binary pump system (Waters 1525), an injector system (Waters 2707), the Waters column heater module WAT038040, a refractive index (RI) detector (Waters 2414), and a dual wavelength absorbance detector (Waters 2487). The samples were loaded on an Aminex HPX-87H cation exchange column (Bio-Rad, München, Germany) coupled to a Micro-guard column (Bio-Rad) and eluted with 5 mM H_2_SO_4_ as the mobile phase at a flow rate of 0.6 mL/min and a column temperature of 45°C. Volumes of 20 µL of sample were used for injection. Succinic acid and malic acid were detected using the dual-wavelength absorbance detector (Waters 2487) while glycerol and ethanol were analysed with the RI detector (Waters 2414). The retention time for malic acid was 9.6 min, for succinic acid 11.9 min, for glycerol 13.5 min, and for ethanol 21.6 min. Data were processed and analysed using the Breeze 2 software (Waters). All reported maximum SA yields were registered at the time points when the HPLC measurements and the respective yield calculations could be done reliably, i.e. between 96 and 144 h of cultivation.

## Results

### The deletion of *MPC3, DIC1*, and *SDH1* genes improved succinic acid production in the DHA-SAT strain

First, we individually deleted *MPC3, MPC1, OAC1, DIC1, SFC1*, and *SDH1* in a *S. cerevisiae* strain previously engineered for SA production from glycerol and CO_2_ via the rTCA pathway. As the current study was conducted in parallel to the study of Malubhoy et al. (2022), we started here with the strain DHA-SAT (i.e. the strain without *PYC2* overexpression), which was first generated by the latter authors. All deletion mutants are listed in Table 1 and their construction is described in Material and Methods. Shake-flask cultures of strain DHA-SAT and the respective mutant strains were performed in a synthetic glycerol medium supplemented with CaCO_3_ to analyse the effects of individual gene deletions on SA production.

**Figure 2.**
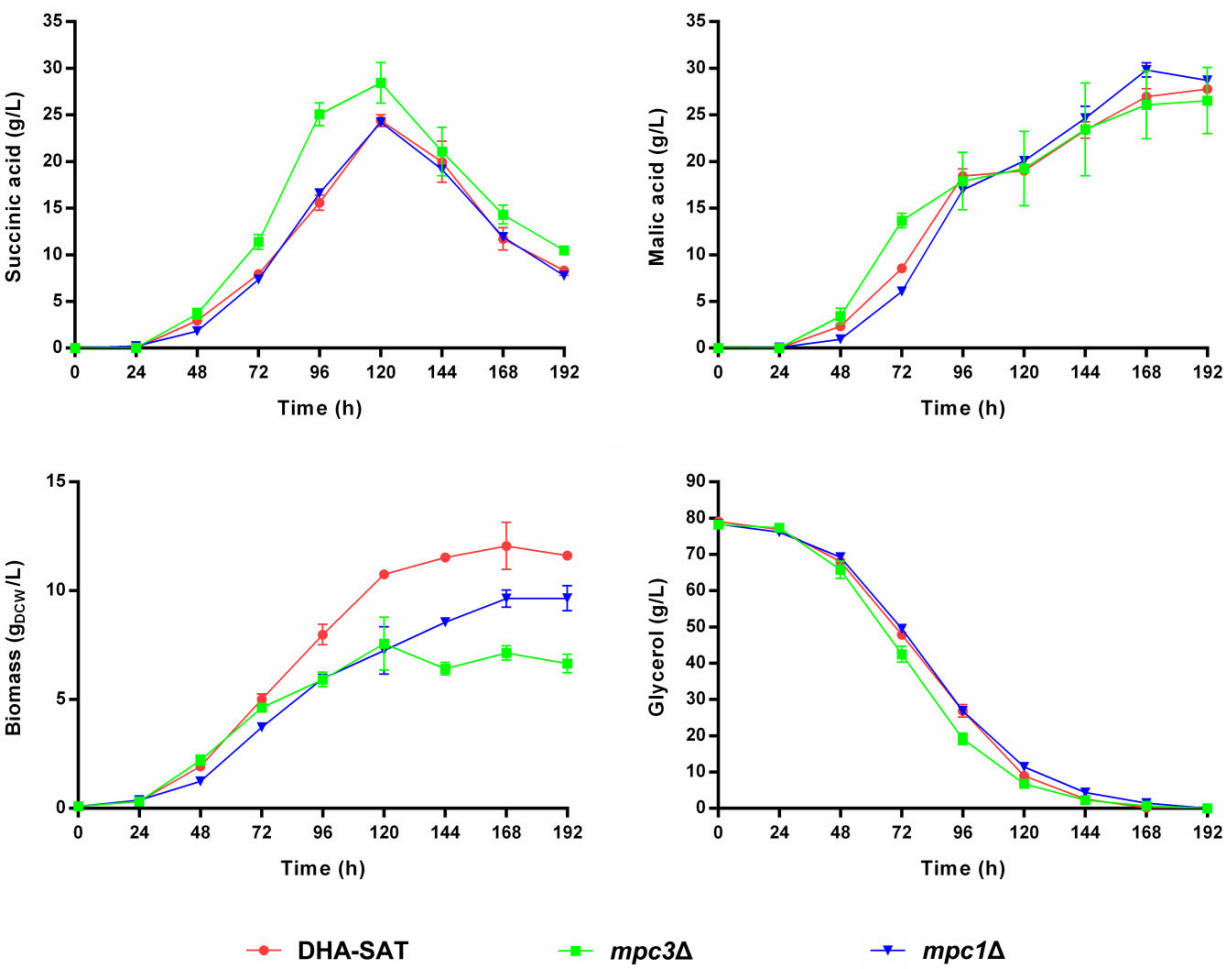
The SA-overproducing *S. cerevisiae* strain DHA-SAT as well as the isogenic deletion strains *mpc3*∆ and *mpc1*∆ cultivated in synthetic glycerol medium using urea as the nitrogen source and buffered with 30 g/L of CaCO_3_ (see composition in Materials and Methods). The cultivations were performed in 500-mL shake flasks filled with 100-mL medium. The initial pH of the medium was 6.0, prior to CaCO_3_ addition. HPLC analysis was used to determine the concentrations of succinic acid, malic acid, and glycerol in the culture supernatant. Biomass accumulation was recorded by optical density measurements at 600 nm (OD_600_). Mean values and standard deviations (SD) were determined from three biological replicates.

As shown in Fig. 2, the *mpc3*∆ strain exhibited a 20% increase in maximum SA yield compared to the DHA-SAT strain. The *mpc3*∆ strain also displayed a significant reduction in biomass accumulation compared to the baseline strain. Interestingly, the *MPC1* deletion did not lead to an improvement in SA production (Fig. 2). However, the *MPC1* deletion had a noticeable impact on growth as the *mpc1*∆ strain had a slightly extended lag phase and accumulated significantly less biomass than the DHA-SAT strain (Fig. 2).

**Figure 3.**
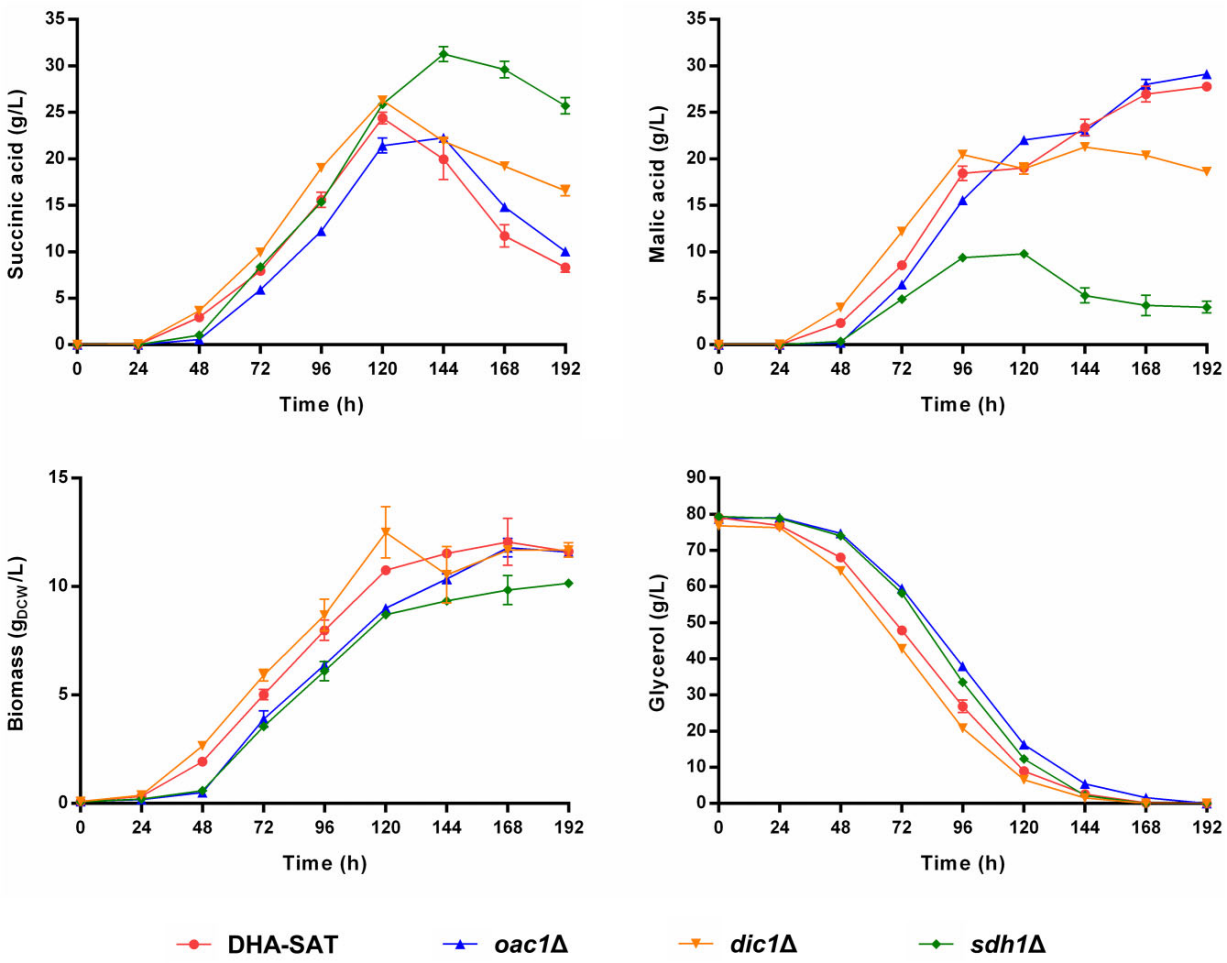
The SA-overproducing *S. cerevisiae* strain DHA-SAT and the isogenic deletion mutants *dic1*∆, *oac1*∆, and *sdh1*∆ cultivated in synthetic glycerol medium using urea as the nitrogen source and buffered with 30 g/L of CaCO_3_ (see composition in Materials and Methods). The cultivations were performed in 500-mL shake flasks filled with 100-mL medium. The initial pH of the medium was 6.0, prior to CaCO_3_ addition. HPLC analysis was used to determine the concentrations of succinic acid, malic acid, and glycerol in the culture supernatant. Biomass accumulation was recorded by measuring optical density at 600 nm (OD_600_). Mean values and SDs were determined from three biological replicates.

As a next step, we investigated the strains with abolished Oac1 (oxaloacetate carrier) or Dic1 (malate and SA carrier) activity. Deletion of *OAC1* led to a slight decrease in the maximum obtained SA titre; however, the maximum SA yield did not significantly differ from the one obtained by the DHA-SAT strain (Fig. 3). The *oac1*∆ strain displayed an extended lag phase compared to the DHA-SAT strain, but both strains accumulated equal amounts of biomass by the end of the cultivation (Fig. 3). The deletion of *DIC1* caused a slight increase in SA production (∼6% increase in maximum SA yield compared to the DHA-SAT strain). Unlike the *OAC1* deletion, the deletion of *DIC1* did not cause a prolonged lag phase (Fig. 3). Additionally, the *DIC1* deletion significantly reduced the re-consumption of SA from the medium upon glycerol exhaustion and prevented the formation of malate during this stage of cultivation. These results indicate that Dic1 indeed participates in the mitochondrial consumption of SA in the DHA-SAT strain, both before and after glycerol exhaustion.

The deletion of *SFC1* did not cause a significant change in the phenotype (Supplementary Fig. S1). The *sfc1*∆ strain reached a similar maximum SA yield and accumulated a similar amount of biomass as the DHA-SAT strain.

As expected, the deletion of *SDH1* caused a significant improvement in SA production; the *sdh1*∆ strain exhibited a ∼17% increase in SA yield over the DHA-SAT strain (Fig. 3). In contrast to all other deletion mutants tested, the *sdh1*∆ strain produced significantly less malate throughout the cultivation when compared with the DHA-SAT strain (Fig. 3). The *sdh1*∆ strain also exhibited an extended lag phase and accumulated less biomass than the DHA-SAT strain.

### Assessment of the promising gene deletions *(mpc3*∆, *dic1*∆, and *sdh1*∆) in the DHA-SAT strain overexpressing *PYC2*

**Figure 4.**
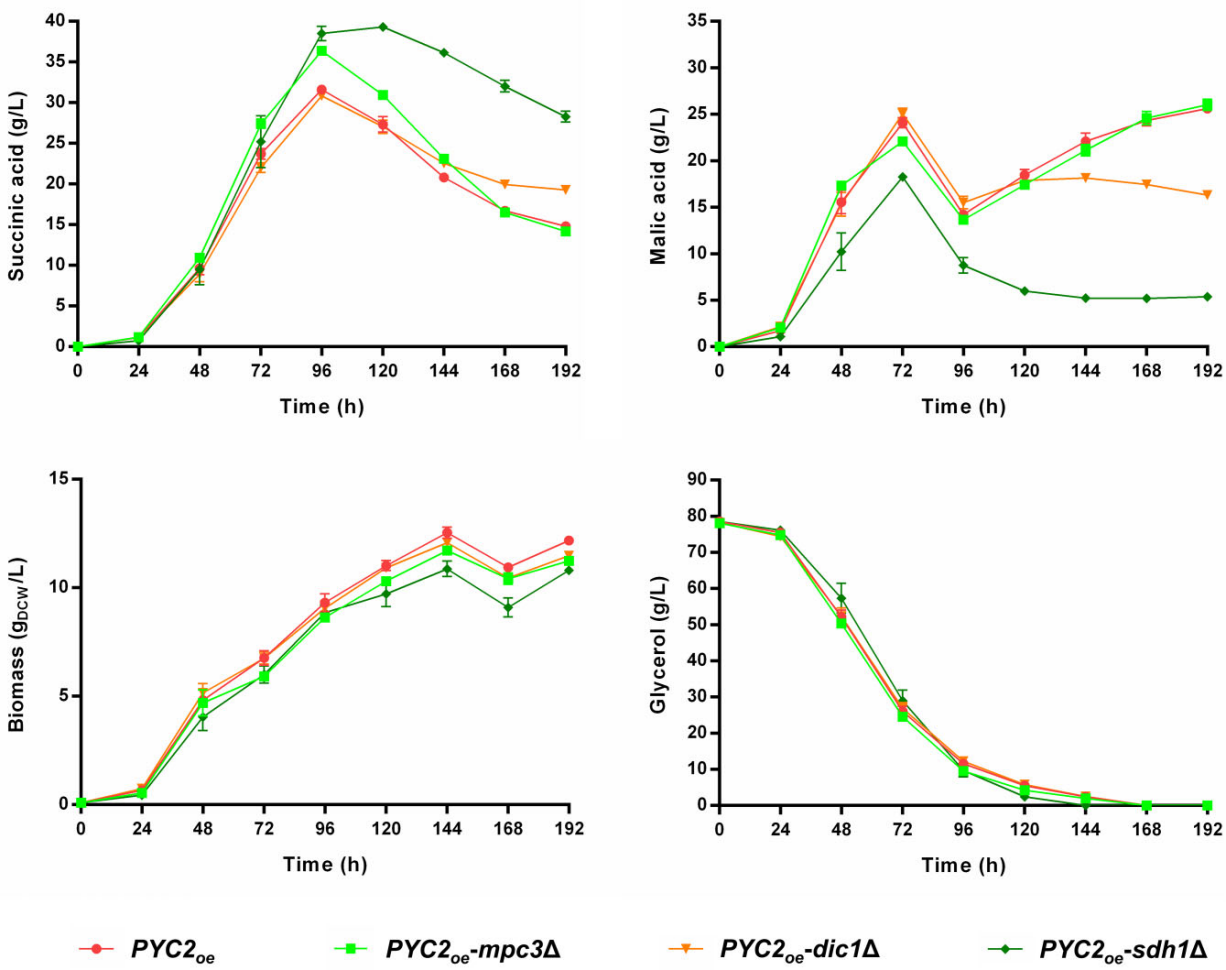
The SA-overproducing *S. cerevisiae* strain *PYC2_oe_* and the isogenic *mpc3*∆, *dic1*∆, and *sdh1*∆ mutant strains cultivated in synthetic glycerol medium using urea as the nitrogen source and buffered with 30 g/L of CaCO_3_ (see composition in Materials and Methods). The cultivations were performed in 500-mL shake flasks filled with 100-mL medium. The initial pH of the medium was 6.0, prior to CaCO_3_ addition. HPLC analysis was used to determine the concentrations of succinic acid, malic acid, and glycerol in the culture supernatant. Biomass accumulation was recorded by measuring optical density at 600 nm (OD_600_). Mean values and SDs were determined from three biological replicates.

In our previous work conducted by Malubhoy et al. (2022), we improved the performance of the DHA-SAT strain (higher SA yield and production rate) by increasing the activity of pyruvate carboxylase in the cytosol, which was achieved by integrating an additional expression cassette for *PYC2*. As a next step, we wanted to check to what extent the SA production by the *PYC2_oe_* strain would benefit from the gene deletions that resulted in SA yield improvements in the strain DHA-SAT (i.e. *MPC3, DIC1*, or *SDH1*). We constructed the respective deletion mutants and compared them with the isogenic *PYC2_oe_* strain in shake flask cultivations in synthetic glycerol media supplemented with CaCO_3_ (Fig. 4).

Deletion of the *MPC3* gene in the *PYC2_oe_* strain background again had a clear positive impact on SA production, causing a ∼13% increase in the maximum SA yield (Fig. 4). The deletion of the *MPC3* gene in the *PYC2_oe_* strain negatively affected growth, although not as severely as in the DHA-SAT strain (Fig. 4). Evidently, the *mpc3* deletion strain managed to direct additional pyruvate into the rTCA pathway, despite the fact that the *PYC2_oe_* strain already had a strong flux through this pathway.

The *PYC2_oe_* strain is assumed to have higher concentrations of SA in the cytosol compared to the DHA-SAT strain. For this reason, we expected that the *DIC1* deletion would have an even higher impact on SA production in the *PYC2_oe_* strain. In contrast to our expectations, the *DIC1* deletion did not improve SA production in this strain background (Fig. 4). However, the *DIC1* deletion reduced the re-consumption of SA from the medium upon glycerol exhaustion, while also preventing malate formation during this stage of cultivation (Fig. 4). These latter results resembled the observations already made in the DHA-SAT strain background.

Deletion of the *SDH1* gene in the *PYC2_oe_* strain again positively affected SA production at the expense of malate production (Fig. 4). A ∼19% increase in maximum SA yield was achieved in the *sdh1* deletion mutant strain compared to the reference *PYC2_oe_* strain (Fig. 4). Moreover, the *PYC2_oe_*-*sdh1*∆ strain accumulated less biomass than the *PYC2_oe_* strain (Fig. 4). However, this difference in growth was not as pronounced as in the DHA-SAT strain background.

**Figure 5.**
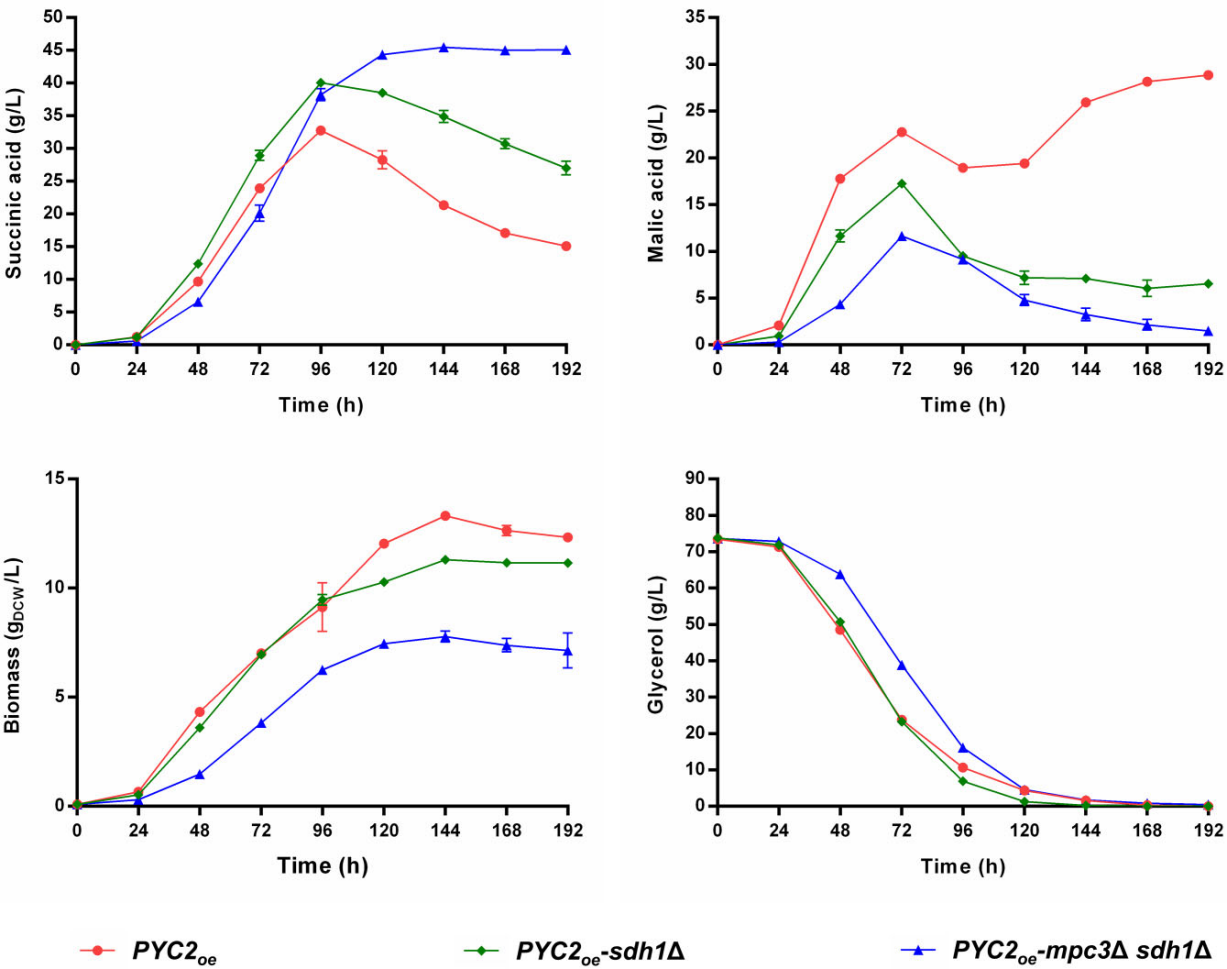
The SA-overproducing *S. cerevisiae* strain *PYC2_oe_* and the isogenic *sdh1*∆ and *mpc3*∆ *sdh1*∆ mutant strains cultivated in synthetic glycerol medium using urea as the nitrogen source and buffered with 30 g/L of CaCO_3_ (see composition in Materials and Methods). The cultivations were performed in 500-mL shake flasks filled with 100-mL medium. The initial pH of the medium prior to CaCO_3_ addition was 6.0. HPLC analysis was used to determine the concentrations of succinic acid, malic acid, and glycerol in the culture supernatant. Biomass accumulation was recorded by measuring optical density at 600 nm (OD_600_). Mean values and SDs were determined from four biological replicates.

Lastly, we decided to combine the two most promising deletions (*mpc3*∆ and *sdh1*∆) in the *PYC2_oe_* strain to test whether they will cause a cumulative effect on SA production. The resulting *PYC2_oe_-mpc3*∆ *sdh1*∆ strain produced 45.5 g/L of SA (at 144 h) and reached a maximum SA yield of 0.66 g_SA_/g_glycerol_ (at 96 h). The yield improvement corresponded to a ∼27% increase over the *PYC2_oe_* strain (Fig. 5). Moreover, the *PYC2_oe_-mpc3*∆ *sdh1*∆ strain accumulated the lowest amounts of malate and biomass in comparison with the other isogenic strains tested.

## Discussion

In biotechnological SA production using microbes, the rTCA pathway is the preferred metabolic route in order to maximize SA yield by fixing CO_2_ (Ahn et al. 2016). To minimize production costs, low external pH is highly favourable, and robust fungal organisms such as *S. cerevisiae* and *Pichia kudriavzevii* represent highly suitable cell factories (Abbott et al. 2009, Tran et al. 2023). Due to ATP constraints, we assume that homofermentative SA production (from glycerol) cannot be run anaerobically, which is in contrast to the well-known ethanol production (from glucose) in yeast. Based on the following considerations, we hypothesize that a portion of the substrate must be respired in the mitochondria to provide the cells with sufficient ATP for SA export, cell growth, and maintenance. In fact, the net ATP yield for the fermentative SA formation from glycerol (or glucose) is zero, particularly when the CO_2_ fixation is carried out by the ATP-consuming pyruvate carboxylase (Fig. 1). On top of that, the export of SA requires energy to be thermodynamically feasible under industrially relevant conditions, i.e. low pH and high extracellular product concentration (de Kok et al. 2012). In this context, the facilitated export of divalent SA anions is considered to be the least energetically expensive type of export thermodynamically feasible in such conditions (de Kok et al. 2012, Rendulić et al. 2022). However, it requires the concurrent export of two protons via H^+^-ATP pumps to maintain cellular pH and charge homeostasis, thus costing two ATP per exported SA molecule (de Kok et al. 2012). For these reasons, efficient fungal-based SA production processes (including commercial production) are carried out in aerobic conditions (Abbott et al. 2009, Jansen et al. 2013, Ahn et al. 2016) to be able to generate the required ATP via mitochondrial respiration.

Although this reasoning makes clear that a certain activity of the oxidative TCA cycle is required for ATP production, it is questionable whether the flux through this pathway in our previously constructed SA producers, DHA-SAT and *PYC2_oe_*, exceeds the required level. Compared to the situation on glucose, the activity of the respiratory metabolism (i.e. the oxTCA cycle and the respiratory chain) is indeed highly upregulated during the growth on respiratory carbon sources such as glycerol, as described by Xiberras et al. (2019). Thus, the mitochondrial activity under non-repressive conditions might be far too high, resulting in suboptimal SA yields.

Consistent with our assumptions, the deletion of *MPC3* had a positive effect on SA yield (Figs 2 and 4) by reducing the influx of pyruvate into the mitochondria. As both SA-producing *S. cerevisiae MPC3* deletion strains still displayed considerable biomass formation, our results suggest that an even stronger reduction in mitochondrial pyruvate import could be sustainable, potentially resulting in an even higher SA yield. The interpretation of our results obtained with the *mpc1*∆ mutant is rather puzzling. According to the literature, the lack of *MPC1* is expected to cause the strongest reduction in mitochondrial uptake of pyruvate because neither of the two active carrier complexes (MPC_OX_ nor MPC_FERM_) can be formed without the Mpc1 subunit (Bender et al. 2015). Our *mpc1*∆ mutant could grow in a synthetic glycerol medium, showing that it obviously still generated sufficient mitochondrial pyruvate to produce energy and branched-chain amino acids, most likely via the net uptake of rTCA intermediates coupled with the malic enzyme activity (Fig. 1). The slow growth of the *mpc1*∆ strain during the early phase of cultivation (Fig. 2) and the increased levels of ethanol registered at 72 h (Supplementary Fig. S2) suggest that the cells indeed experienced a lack of mitochondrial pyruvate uptake as well as an accumulation of pyruvate in the cytosol. Thus, it was surprising that there was no increase in SA production compared to the baseline DHA-SAT strain. Perhaps the complete lack of mitochondrial pyruvate uptake triggered a strong rewiring of the central carbon metabolism to sustain growth, which consequently nullified the increased supply of cytosolic pyruvate for SA production. Hypothetically, a strong upregulation of alternative metabolic routes such as the PDH bypass, the glyoxylate cycle, the mitochondrial uptake of rTCA intermediates, and the malic enzyme reaction might have caused such a phenomenon. The remaining question would be why such a rewiring did not take place in our *mpc3*∆ strain. Overall, more research is required to better understand the interplay between the mitochondrial transport of pyruvate (or lack thereof) and the regulatory mechanisms which control the central carbon metabolism in glycerol-grown and SA-overproducing *S. cerevisiae* cells.

By deleting *OAC1* or *DIC1*, the current study attempted to limit the import of cytosolic rTCA intermediates into the mitochondria, thereby limiting the replenishment of the oxTCA cycle with C_4_ compounds. Provided that the formation of acetyl-CoA is not rate-controlling for the oxTCA, our approach might reduce the amount of available mitochondrial oxaloacetate for the dissimilation of acetyl-CoA and thus the loss of carbon ending up in CO_2_. The observed prolonged lag phase of the *oac1*∆ strain, which occurred upon transferring the strain from the glucose-based preculture medium into the glycerol-based main culture medium, may indeed be explained in this way. However, the *oac1*∆ strain eventually reached similar growth rates as the reference strain (DHA-SAT) and did not show any improvements in the SA yield. Thus, the activity of the remaining Dic1 transporter could have compensated for the lack of Oac1. In contrast to the deletion of *OAC1*, the deletion of *DIC1* in the DHA-SAT strain slightly improved the SA yield. The fact that the *dic1* deletion strain did not display any impediments in cell growth might indicate that the improved SA production was the result of a reduced dissimilation of cytosolic malate and SA into CO_2_ (via the mitochondrial malic enzyme and the PDH complex) rather than merely a limitation in anaplerotic pathways.

Another interesting observation based on our *dic1*∆ mutants (Figs 3 and 4) is that Dic1 obviously participates in the consumption of extracellular SA and the buildup of malate in the period after glycerol is consumed. The uptake of extracellular SA into the cytosol upon glycerol exhaustion can be attributed to the dicarboxylate plasma membrane transporter Dct-02 (Rendulić et al. 2022). Dic1 seems to import cytosolic SA into the mitochondria, where it is oxidized into fumarate via the SDH complex and further converted into malate via the fumarase Fum1. The results further suggest that the obtained mitochondrial malate is exported back into the cytosol (possibly also via Dic1), and subsequently into the medium via Dct-02. The complete process involving the activities of Dct-02, Dic1, the SDH complex, and Fum1 channels electrons via FADH_2_ into the respiratory chain and thus may provide the cells with an alternative source of energy upon depletion of glycerol.

Overall, our attempts to limit the import of rTCA intermediates (C_4_) into the mitochondria did not lead to improvements in SA yield that were as high as the ones obtained by limiting the mitochondrial pyruvate uptake. This may be partially explained by the fact that the standalone deletion of *OAC1* can be compensated by *DIC1* and vice versa. Notably, the double deletion of *OAC1* and *DIC1* was previously reported to disable the growth of *S. cerevisiae* on non-fermentable carbon sources in synthetic media due to a complete abolishment of anaplerosis (Palmieri et al. 1999). Thus, future metabolic engineering endeavours may focus on simultaneously tuning down the activities of Oac1 and Dic1, rather than completely abolishing them.

In order to achieve optimal SA yields, one has to make sure that, in addition to carbon, unnecessary loss of electrons in respiration is avoided. One of the objectives of this study was to investigate the existence of a hypothetical succinate-fumarate shuttle (mediated by Sfc1), which was considered to transfer electrons from cytosolic NADH into the mitochondrial respiratory chain (via SDH). Our results showed that this potential shuttle is not relevant, at least when the genetic constitution of the strain and the conditions used are considered. One reason might be that the fumarate concentration in mitochondria is not high enough to drive this shuttle. It might also be that other, more obvious, routes of oxidizing cytosolic NADH, such as the external mitochondrial NADH dehydrogenases Nde1 and Nde2 (Bakker et al. 2001), lead to a loss of electrons from cytosolic NADH and reduce SA yield.

Disruption of the mitochondrial SDH complex by knocking out one of its subunits represents a common and straightforward strategy to induce SA overproduction via the oxTCA cycle in *S. cerevisiae* and other yeasts (Raab et al. 2010, Ito et al. 2014, Gao et al. 2016, Xi et al. 2021). In strains exhibiting the rTCA pathway in the cytosol, the deletion of *SDH1* can contribute to an increase in SA production in two ways: (i) by blocking the oxidation of mitochondrial SA and leading to a net SA export to the cytosol and, subsequently, to the culture medium, and (ii) by preventing mitochondrial dissimilation of the SA that is generated in the cytosol by the rTCA pathway and imported to mitochondria via the transporter Dic1 and/or Sfc1. In the current study, the *sdh1*∆ represented a useful control. In fact, the deletion of the two genes that encode mitochondrial succinate transporters (*DIC1* and *SFC1*) caused little to no improvement in SA production (Figs 3, 4, and Supplementary Fig. S1), while the deletion of *SDH1* resulted in a significant improvement. Taken together, these results suggest that the additional SA obtained by deleting *SDH1* rather originates from the oxidative than from the reductive SA production route. Still, this is not considered counterproductive for our efforts to increase the SA production via the CO_2_-fixing rTCA pathway because the *SDH1* deletion is not supposed to increase the flux of carbon into the mitochondria.

Another consequence of the *SDH1* deletion observed in the current study is a reduced accumulation of malate in the culture medium (Figs 3 and 4). In fact, the observed decrease in malate yield corresponded to the observed increase in SA yield (in mol_acid_/mol_glycerol_), when comparing the *PYC2_oe_*-*sdh1*Δ and *PYC2_oe_* strains (Supplementary Fig. S3). As the lack of Sdh1 abolished the mitochondrial conversion of SA into fumarate, malate, and oxaloacetate (Fig. 1), the *sdh1* deletion mutants must utilize alternative means to provide the acceptor for acetyl-CoA in the oxTCA, and thus oxaloacetate, malate, and/or fumarate must be imported from the cytosol. The lower concentration of malate detected for the *sdh1* deletion mutants equipped with the rTCA pathway could be an indication that these strains indeed make use of this opportunity. A reduced accumulation of malate inside the cell can be considered a benefit for SA production via yeast, particularly since Dct-02 also exports malate alongside SA into the culture medium (Rendulić et al. 2022).

In conclusion, we were able to further increase the maximum SA yield compared to our previously published strain by targeting mitochondrial transporters and/or the SDH complex. The highest improvement in yield (27%) was achieved by combining the benefits of deleting *MPC3* (improved cytosolic rTCA flux) with the benefits of deleting *SDH1* (increase in cytosolic SA and a drop in cytosolic malate). To our knowledge, the obtained maximum SA yield (0.66 g/g) represents the highest SA yield that was obtained thus far using yeasts (Table 3). Notably, this result was achieved using shake-flasks without the particular effort to optimize cultivation conditions. Known process engineering techniques could therefore be exploited to achieve potentially even better key performance parameters with this strain. For example, nitrogen-limitation could be applied to uncouple growth from SA production, thereby further reducing biomass formation and maximizing SA yield (Liu et al. 2021). Although the results have been obtained here with glycerol as a carbon source, the metabolic engineering strategies presented in our work might also support future efforts to improve the SA production from sugars. Apart from the direct improvements shown here, the results of the current study can guide us even further towards improvements in SA production with yeast-based production processes. We consider the further reduction of mitochondrial pyruvate import combined with a further reduction in mitochondrial import of rTCA intermediates highly attractive. This might also be combined with the reduction of electron transfer from cytosolic NADH to the mitochondrial electron transport chain. In general, it will be challenging to predict the optimal expression levels of gene targets involved, and a combinatorial approach is considered most promising.

**Table 3.** Reported fermentation performance of engineered SA-producing *S. cerevisiae, Issatchenkia orientalis* (also known as *Pichia kudriavzevii*), and *Yarrowia lipolytica* strains.

Yeast strain	Cultivation type	Max. yield (g/g)	Max. titre (g/L)	Study
*S. cerevisiae* AH22ura3 Δ*sdh2*Δ*sdh1*Δ*idh1*Δ*idp1*	Shake-flask (batch); glucose	0.07	3.62	Raab et al. (2010)
*S. cerevisiae* SUC-632	Bioreactor (fed-batch); glucose	0.52	ND	Jansen et al. (2013)
*S. cerevisiae* PMCFfg	Shake-flask (batch); glucose	0.21	10.0	Yan et al. (2014)
*S. cerevisiae UBR2_CBS_*-DHA-SA-*An*DCT-02	Shake-flask (batch); glycerol	0.22	10.7	Xiberras et al. (2020)
*S. cerevisiae PYC2_oe_*	Shake-flask (batch); glycerol	0.52	32.9	This study
*S. cerevisiae PYC2_oe_-mpc3*Δ *sdh1*Δ	Shake-flask (batch); glycerol	0.66	45.5	This study
*I. orientalis* Io − ura3−+−SA	Shake-flask (batch); glucose	0.12	11.6	Xiao et al. (2014)
*I. orientalis* g3473∆/PaGDH-DAK/g3837∆	Bioreactor (fed-batch); glucose and glycerol	0.65	109.5	Tran et al. (2023)
*Y. lipolytica* PGC01003	Bioreactor (fed-batch); glycerol	0.40	160.2	Gao et al. (2016)
*Y. lipolytica* PGC202	Bioreactor (fed-batch); glycerol	0.53	110.7	Cui et al. (2017)
*Y. lipolytica* PGC202	Bioreactor (batch); glucose	0.61	53.6	Yu et al. (2018)

ND: not determined.

## Supplementary Material

foae009_Supplemental_Files
