# Supplementary material for: Mitochondrial membrane transporters as attractive targets for the fermentative production of succinic acid from glycerol in Saccharomyces cerevisiae
Source: FEMS Yeast Res. 2024 Apr 8;24:foae009. doi: 10.1093/femsyr/foae009 (PMC11014245; doi:10.1093/femsyr/foae009)
Supplement: foae009_Supplemental_Files [file foae009_supplemental_files.zip › Rendulic et al 2024 FEMS-supplementary data-PDF.pdf]

SUPPLEMENTARY MATERIAL

Table S1. Primers and templates used for amplification of disruption cassettes.

Construct	Position of integration	Amplified fragment	Template	Primer number	Sequence
mpc3::loxP-ble-loxP	*MPC3* / YGR243W	loxP-P_{TEF1}-ble-T_{TEF1}-loxP	pUG66	1533	AGCATTCAAGACACATAGAAACACAAACCTATATTTTTACCAGCTGAAGCTTCGTACGC
				1534	ATGCGAGTTCAGGAACATATTATCGTTTACGTAAGCCGCATAGGCCACTAGTGGATCTG
mpc1::loxP-ble-loxP	*MPC1* / YGL080W	loxP-P_{TEF1}-ble-T_{TEF1}-loxP	pUG66	1529	ATATATACGTATAGATTTTATTGCACTGTGATCAAAAAGACCAGCTGAAGCTTCGTACGC
				1530	ATCTAGTCACCTACTTCAGGTTCTTAGACTGCTCGTTCGCATAGGCCACTAGTGGATCTG
oac1::loxP-ble-loxP	*OAC1* / YKL120W	loxP-P_{TEF1}-ble-T_{TEF1}-loxP	pUG66	1403	AGACACAAGCACATCTCATCGAATTATATCGTAAGCAAATCCAGCTGAAGCTTCGTACGC
				1404	TGGCCAATGAATGAAACTTCAAACCTCGGAGTTTGTTATGGGAACGCATAGGCCACTAGTGGATCTG
dic1::loxP-ble-loxP	*DIC1* / YLR348C	loxP-P_{TEF1}-ble-T_{TEF1}-loxP	pUG66	1708	GTAGAGGTTCGTTTCTCTTGCTCTGAAAGTGTCGAAAAGATAACGCAACAGCTGGACGGCCCAGCTGAAGCTTCGTACGC
				1709	TACTCTTCTTGCTTTCTTTATTTGCTATGTATCTTTATGTTTATATGTATATAAATCTGCCGCATAGGCCACTAGTGGATCTG
sfc1::loxP-ble-loxP	*SFC1* / YJR095W	loxP-P_{TEF1}-ble-T_{TEF1}-loxP	pUG66	1704	AGAAAGAAGTTTATATTAGTTTTAGCCGTAAGATAACATAACAAAGAAGAAGAAAGAAAACCAGCTGAAGCTTCGTACGC
				1705	GATCTTCTATTCTATTTCTATTTTTTCTTTATTTCATTTTGTAGTCCCATTGTTCATCATCGCATAGGCCACTAGTGGATCTG

sdh1::loxP-ble-loxP	*SDH1* / YKL148C	loxP-P_{TEF1}-ble-T_{TEF1}-loxP	pUG66	1243	AGAAAGAAAAAAATCCAATTTCATAGTACGAAGAAGAACGAGAATAAAGCCAGCTGAAGCTTCGTACGC
				1244	AAAGAAGAGTATGATATTCTTTTCCGTAAAATACAATGAGGTTCAAACGCATAGGCCACTAGTGGATCTG
mpc3::hphMX	*MPC3* / YGR243W	P_{TEF1}-hphMX-T_{TEF1}	pAG32	1533	AGCATTCAAGACACATAGAAACACAAACCTATATTTTTACCAGCTGAAGCTTCGTACGC
				1534	ATGCGAGTTCAGGAACATATTATCGTTTACGTAAGCCGCATAGGCCACTAGTGGATCTG
dic1::hphMX	*DIC1* / YLR348C	P_{TEF1}-hphMX-T_{TEF1}	pAG32	1708	GTAGAGGTTCGTTTCTCTTGCTCTGAAAGTGTCGAAAAGATAACGCAACAGCTGGACGGCCCAGCTGAAGCTTCGTACGC
				1709	TACTCTTCTTGCTTTCTTTATTTGCTATGTATCTTTATGTTTATATGTATATAAATCTGCCGCATAGGCCACTAGTGGATCTG
sdh1::hphMX	*SDH1* / YKL148C	P_{TEF1}-hphMX-T_{TEF1}	pAG32	1243	AGAAAGAAAAAAATCCAATTTCATAGTACGAAGAAGAACGAGAATAAAGCCAGCTGAAGCTTCGTACGC
				1244	AAAGAAGAGTATGATATTCTTTTCCGTAAAATACAATGAGGTTCAAACGCATAGGCCACTAGTGGATCTG
sdh1::loxP-natMX-loxP	*SDH1* / YKL148C	loxP-P_{TEF1}-natMX-T_{TEF1}-loxP	pUG74	1243	AGAAAGAAAAAAATCCAATTTCATAGTACGAAGAAGAACGAGAATAAAGCCAGCTGAAGCTTCGTACGC
				1244	AAAGAAGAGTATGATATTCTTTTCCGTAAAATACAATGAGGTTCAAACGCATAGGCCACTAGTGGATCTG

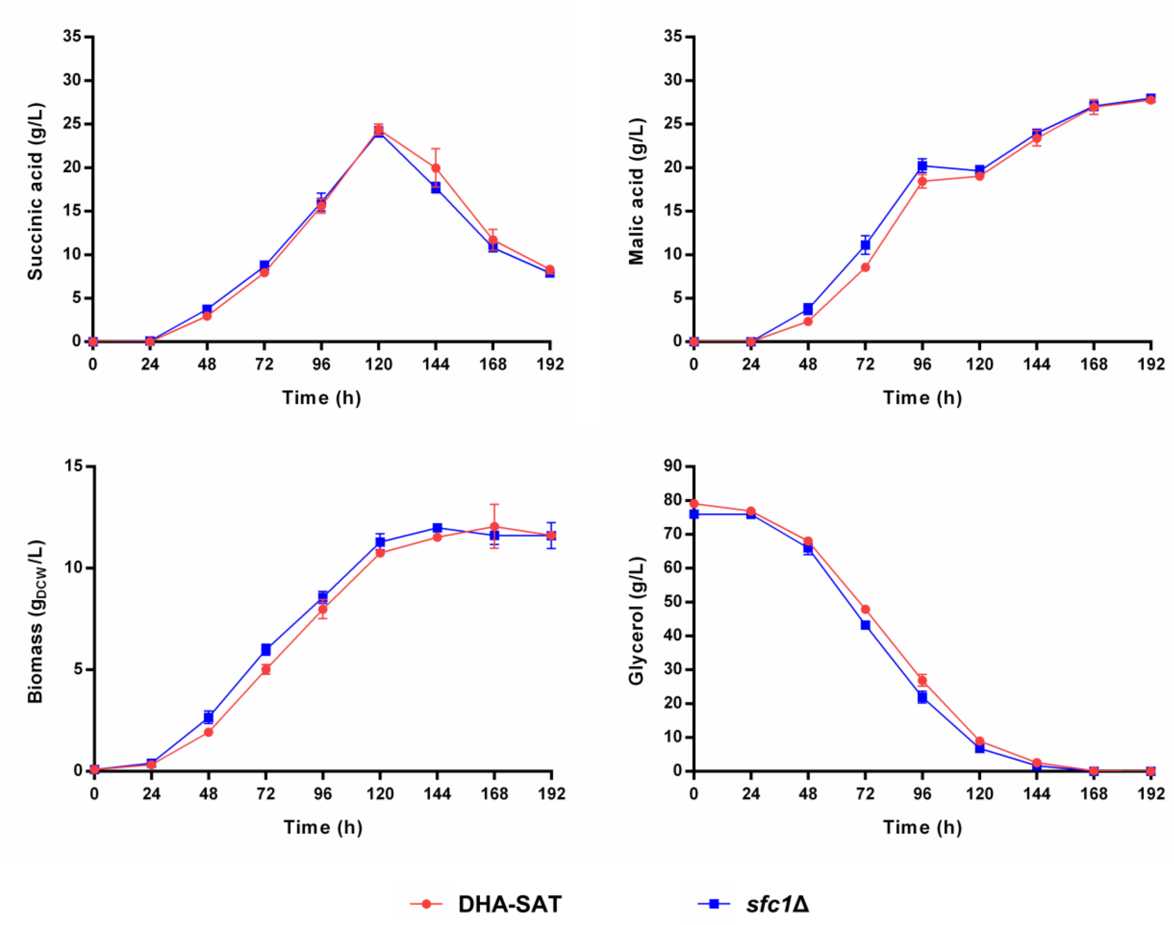

Figure S1. The SA-overproducing *Saccharomyces cerevisiae* strain DHA-SAT and the isogenic *sfc1*Δ mutant cultivated in synthetic glycerol medium using urea as the nitrogen source and buffered with 30 g/L of $CaCO_3$ (see composition in materials and methods). The cultivations were performed in 500 mL shake flasks filled with 100 mL medium. The initial pH of the medium was 6.0, prior to $CaCO_3$ addition. HPLC analysis was used to determine the concentrations of succinic acid, malic acid, and glycerol in the culture supernatant. Biomass accumulation was recorded by measuring optical density at 600 nm (OD_{600}). Mean values and standard deviations were determined from three biological replicates.

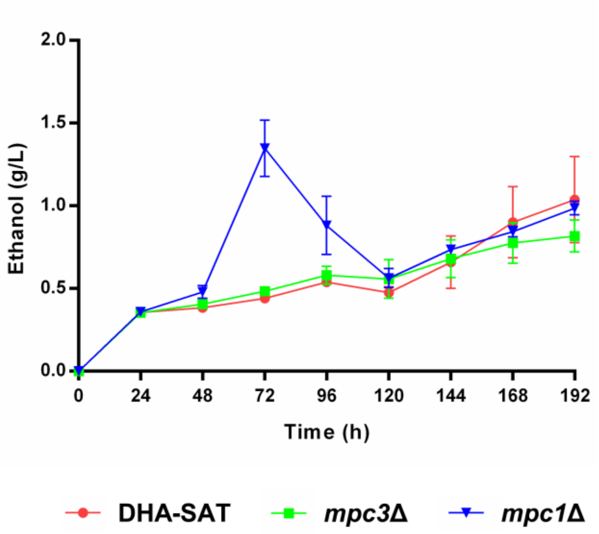

Figure S2. Ethanol production by the SA-overproducing *Saccharomyces cerevisiae* strain DHA-SAT and the isogenic *mpc3*Δ and *mpc1*Δ mutants cultivated in synthetic glycerol medium using urea as the nitrogen source and buffered with 30 g/L of $CaCO_3$ (see composition in materials and methods). The cultivations were performed in 500 mL shake flasks filled with 100 mL medium. The initial pH of the medium was 6.0, prior to $CaCO_3$ addition. HPLC analysis was used to determine the concentrations of ethanol. Mean values and standard deviations were determined from three biological replicates.

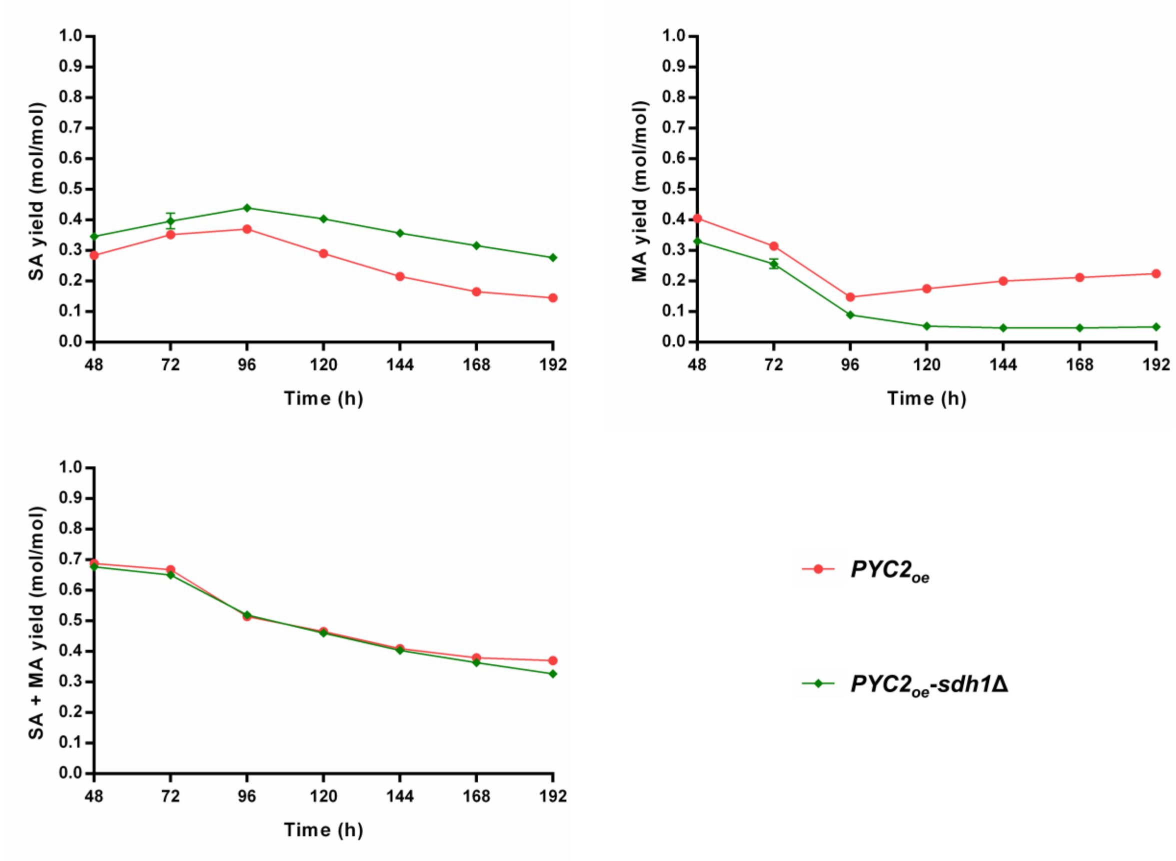

Figure S3. Separate and combined SA and malic acid (MA) yields ($mol_{acid}/mol_{glycerol}$) obtained by the SA-overproducing *Saccharomyces cerevisiae* strain *PYC2*$_{oe}$ and the isogenic *sdh1*Δ mutant strain cultivated in synthetic glycerol medium using urea as the nitrogen source and buffered with 30 g/L of $CaCO_3$ (see composition in materials and methods). Mean values and standard deviations were determined from three biological replicates.
