# Supplementary material for: Mitochondrial membrane transporters as attractive targets for the fermentative production of succinic acid from glycerol in Saccharomyces cerevisiae
Source: FEMS Yeast Res. 2024 Apr 8;24:foae009. doi: 10.1093/femsyr/foae009 (PMC11014245; doi:10.1093/femsyr/foae009)
Supplement: foae009_Supplemental_Files [file foae009_supplemental_files.zip › Rendulic et al 2024 FEMS-supplementary data-WORD.docx]

**SUPPLEMENTARY MATERIAL**

**Table S1.** Primers and templates used for amplification of disruption cassettes.

| **Construct** | **Position of integration** | **Amplified fragment** | **Template** | **Primer number** | **Sequence** |
| --- | --- | --- | --- | --- | --- |
| ***mpc3::loxP-ble-loxP*** | *MPC3* / YGR243W | loxP-P_TEF1_-ble-T_TEF1_-loxP | pUG66 | 1533 | AGCATTCAAGACACATAGAAACACAAACCTATATTTTTACCAGCTGAAGCTTCGTACGC |
|  |  |  |  | 1534 | ATGCGAGTTCAGGAACATATTATCGTTTACGTAAGCCGCATAGGCCACTAGTGGATCTG |
| ***mpc1::loxP-ble-loxP*** | *MPC1* / YGL080W | loxP-P_TEF1_-ble-T_TEF1_-loxP | pUG66 | 1529 | ATATATACGTATAGATTTTATTGCACTGTGATCAAAAAGACCAGCTGAAGCTTCGTACGC |
|  |  |  |  | 1530 | ATCTAGTCACCTACTTCAGGTTCTTAGACTGCTCGTTCGCATAGGCCACTAGTGGATCTG |
| ***oac1::loxP-ble-loxP*** | *OAC1* / YKL120W | loxP-P_TEF1_-ble-T_TEF1_-loxP | pUG66 | 1403 | AGACACAAGCACATCTCATCGAATTATATCGTAAGCAAATCCAGCTGAAGCTTCGTACGC |
|  |  |  |  | 1404 | TGGCCAATGAATGAAACTTCAAACCTCGGAGTTTGTTATGGGAACGCATAGGCCACTAGTGGATCTG |
| ***dic1::loxP-ble-loxP*** | *DIC1* / YLR348C | loxP-P_TEF1_-ble-T_TEF1_-loxP | pUG66 | 1708 | GTAGAGGTTCGTTTCTCTTGCTCTGAAAGTGTCGAAAAGATAACGCAACAGCTGGACGGCCCAGCTGAAGCTTCGTACGC |
|  |  |  |  | 1709 | TACTCTTCTTGCTTTCTTTATTTGCTATGTATCTTTATGTTTATATGTATATAAATCTGCCGCATAGGCCACTAGTGGATCTG |
| ***sfc1::loxP-ble-loxP*** | *SFC1* / YJR095W | loxP-P_TEF1_-ble-T_TEF1_-loxP | pUG66 | 1704 | AGAAAGAAGTTTATATTAGTTTTAGCCGTAAGATAACATAACAAAGAAGAAGAAAGAAAACCAGCTGAAGCTTCGTACGC |
|  |  |  |  | 1705 | GATCTTCTATTCTATTTCTATTTTTTCTTTATTTCATTTTGTAGTCCCATTGTTCATCATCGCATAGGCCACTAGTGGATCTG |
| ***sdh1::loxP-ble-loxP*** | *SDH1* / YKL148C | loxP-P_TEF1_-ble-T_TEF1_-loxP | pUG66 | 1243 | AGAAAGAAAAAAATCCAATTTCATAGTACGAAGAAGAACGAGAATAAAGCCAGCTGAAGCTTCGTACGC |
|  |  |  |  | 1244 | AAAGAAGAGTATGATATTCTTTTCCGTAAAATACAATGAGGTTCAAACGCATAGGCCACTAGTGGATCTG |
| ***mpc3::hphMX*** | *MPC3* / YGR243W | P_TEF1_-hphMX-T_TEF1_ | pAG32 | 1533 | AGCATTCAAGACACATAGAAACACAAACCTATATTTTTACCAGCTGAAGCTTCGTACGC |
|  |  |  |  | 1534 | ATGCGAGTTCAGGAACATATTATCGTTTACGTAAGCCGCATAGGCCACTAGTGGATCTG |
| ***dic1::hphMX*** | *DIC1* / YLR348C | P_TEF1_-hphMX-T_TEF1_ | pAG32 | 1708 | GTAGAGGTTCGTTTCTCTTGCTCTGAAAGTGTCGAAAAGATAACGCAACAGCTGGACGGCCCAGCTGAAGCTTCGTACGC |
|  |  |  |  | 1709 | TACTCTTCTTGCTTTCTTTATTTGCTATGTATCTTTATGTTTATATGTATATAAATCTGCCGCATAGGCCACTAGTGGATCTG |
| ***sdh1::hphMX*** | *SDH1* / YKL148C | P_TEF1_-hphMX-T_TEF1_ | pAG32 | 1243 | AGAAAGAAAAAAATCCAATTTCATAGTACGAAGAAGAACGAGAATAAAGCCAGCTGAAGCTTCGTACGC |
|  |  |  |  | 1244 | AAAGAAGAGTATGATATTCTTTTCCGTAAAATACAATGAGGTTCAAACGCATAGGCCACTAGTGGATCTG |
| ***sdh1::loxP-natMX-loxP*** | *SDH1* / YKL148C | loxP-P_TEF1_-natMX-T_TEF1_-loxP | pUG74 | 1243 | AGAAAGAAAAAAATCCAATTTCATAGTACGAAGAAGAACGAGAATAAAGCCAGCTGAAGCTTCGTACGC |
|  |  |  |  | 1244 | AAAGAAGAGTATGATATTCTTTTCCGTAAAATACAATGAGGTTCAAACGCATAGGCCACTAGTGGATCTG |


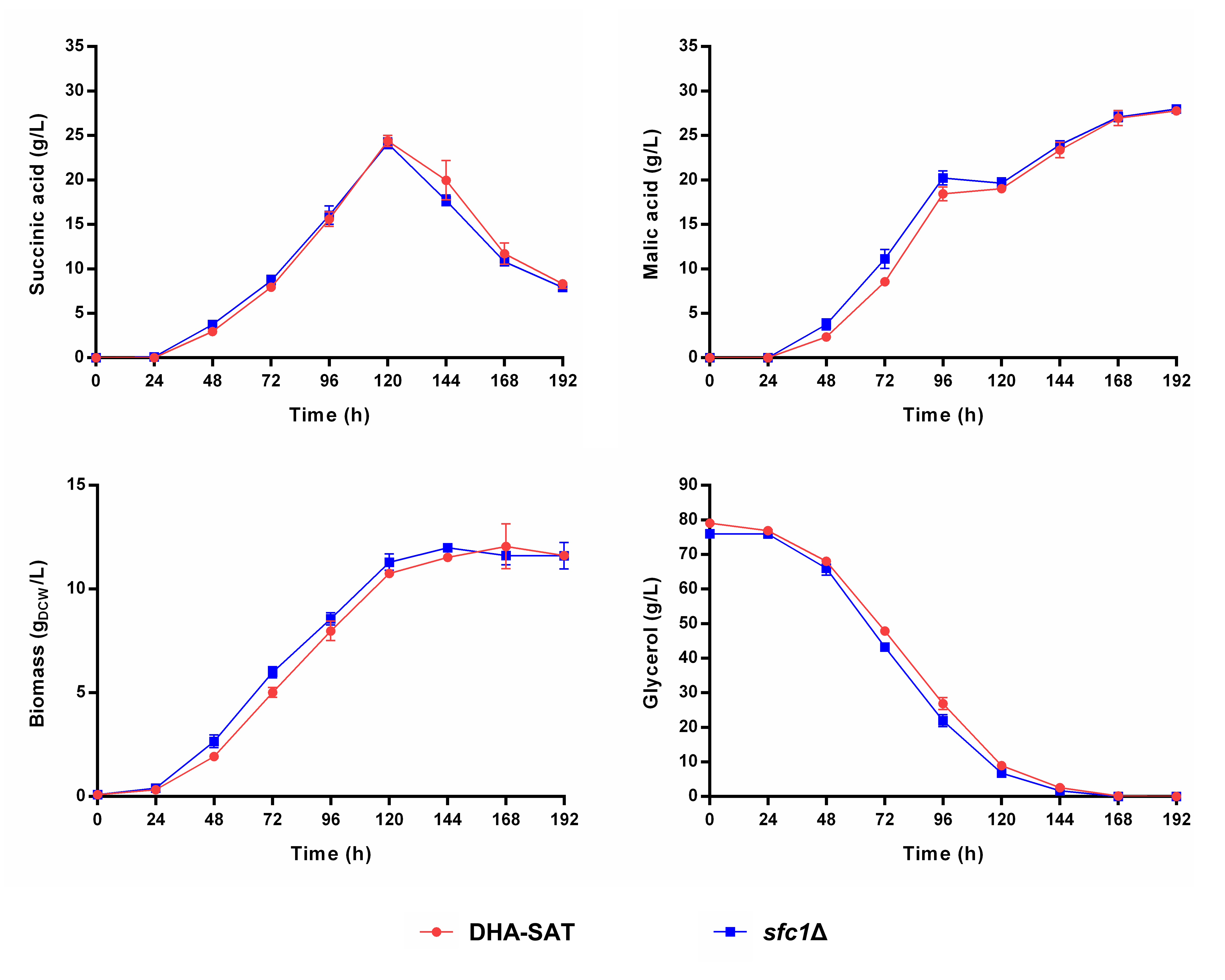


**Figure S1.** The SA-overproducing *Saccharomyces cerevisiae* strain DHA-SAT and the isogenic *sfc1*∆ mutant cultivated in synthetic glycerol medium using urea as the nitrogen source and buffered with 30 g/L of CaCO_3_ (see composition in materials and methods). The cultivations were performed in 500 mL shake flasks filled with 100 mL medium. The initial pH of the medium was 6.0, prior to CaCO_3_ addition. HPLC analysis was used to determine the concentrations of succinic acid, malic acid, and glycerol in the culture supernatant. Biomass accumulation was recorded by measuring optical density at 600 nm (OD_600_). Mean values and standard deviations were determined from three biological replicates.


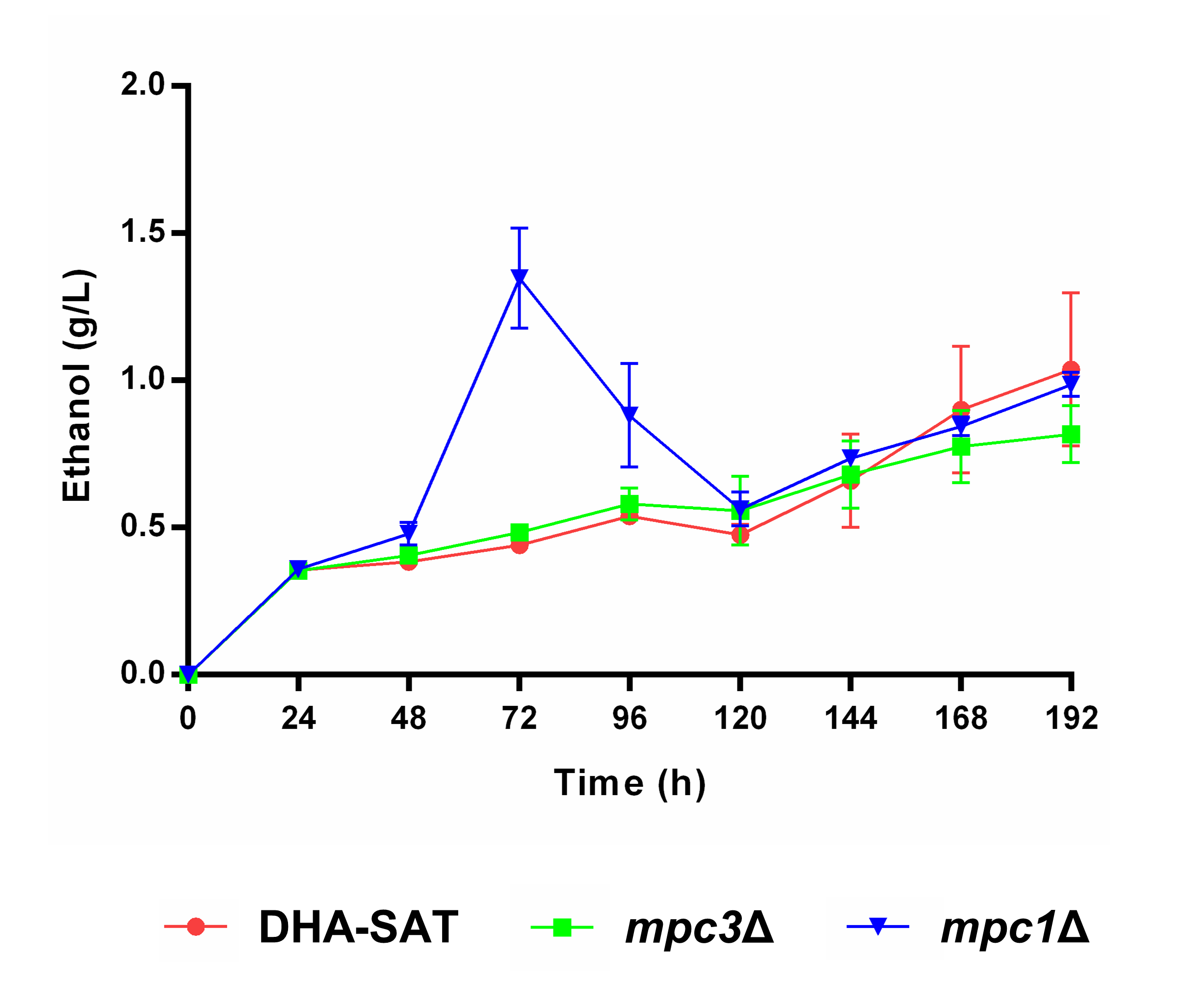


**Figure S2.** Ethanol production by the SA-overproducing *Saccharomyces cerevisiae* strain DHA-SAT and the isogenic *mpc3*∆ and *mpc1*∆ mutants cultivated in synthetic glycerol medium using urea as the nitrogen source and buffered with 30 g/L of CaCO_3_ (see composition in materials and methods). The cultivations were performed in 500 mL shake flasks filled with 100 mL medium. The initial pH of the medium was 6.0, prior to CaCO_3_ addition. HPLC analysis was used to determine the concentrations of ethanol. Mean values and standard deviations were determined from three biological replicates.


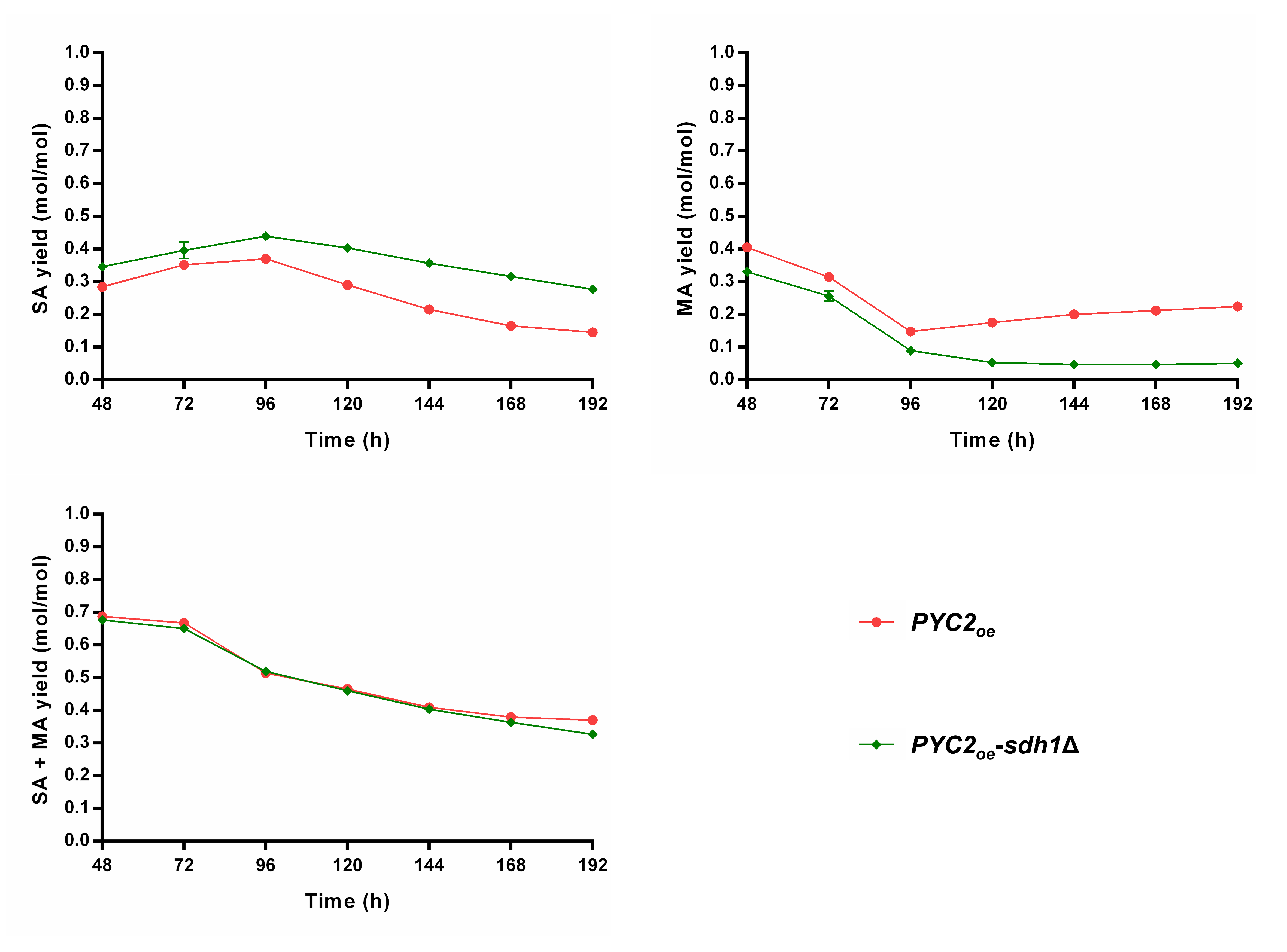


**Figure S3.** Separate and combined SA and malic acid (MA) yields (mol_acid_/mol_glycerol_) obtained by the SA-overproducing *Saccharomyces cerevisiae* strain *PYC2_oe_* and the isogenic *sdh1*∆ mutant strain cultivated in synthetic glycerol medium using urea as the nitrogen source and buffered with 30 g/L of CaCO_3_ (see composition in materials and methods). Mean values and standard deviations were determined from three biological replicates.
